# Antiplatelet Therapy in the Secondary Prevention of Non-cardioembolic Ischemic Stroke and Transient Ischemic Attack: A Mini-Review

**DOI:** 10.3389/fneur.2021.626106

**Published:** 2021-02-25

**Authors:** Martin Vališ, Blanka Klímová, Michal Novotný, Roman Herzig

**Affiliations:** Department of Neurology, Faculty of Medicine and University Hospital Hradec Kralove, Charles University, Hradec Kralove, Czechia

**Keywords:** ischemic stroke, transient ischemic attack, aspirin, clopidogrel, cilostazol, ticagrelor, dipyridamole, antiplatelet therapy

## Abstract

The aim of this mini-review is to discuss the main antiplatelet agents that have been successfully used in the secondary prevention of non-cardioembolic ischemic stroke and transient ischemic attacks (TIA). The methodology is based on a literature review of available peer-reviewed English studies listed in PubMed. The findings reveal that aspirin remains a reliable antiplatelet agent in the secondary prevention of acute non-cardioembolic ischemic stroke and TIA. Nevertheless, currently, there are also other agents, i.e., ticagrelor, clopidogrel, and cilostazol, that can be applied. In addition, the results indicate that time is significant not only in severe stroke but also in non-severe stroke and TIA, which suggests that antiplatelet therapy should be applied within 24 h after the first symptoms because early treatment can lead to an improvement in neurological outcomes and reduce the chance of an early subsequent stroke.

## Introduction

Strokes are among the major causes of morbidity, mortality, and long-term disability (1). As stated by Béjot et al. (2), monthly mortality from strokes in Europe ranges from 13 to 35%. In addition, in European Union countries, the number of patients with strokes is expected to grow by 27% between 2017 and 2047. The reason for this is that there has been an increase in aging population groups and better survival rates after a stroke (3).

The most frequent type of stroke, comprising on average between 80 and 90% of all strokes, is ischemic stroke (4). This type of stroke results from the occlusion of the artery that supplies blood to the brain. The occlusion decreases blood flow and oxygen to the brain, contributing to harm or death of brain cells. If the circulation is not reestablished quickly, the brain damage can be permanent (5). The severity of ischemic stroke ranges from clinically mild (i.e., a minor stroke or transient ischemic attack [TIA]) to very severe (i.e., a major ischemic stroke), but the underlying causes are identical (6). The initial manifestations of ischemic stroke and TIA are often followed by recurrent vascular events, including recurrent strokes (7).

At present, antiplatelet therapy is crucial in the management of non-cardioembolic ischemic stroke and TIA, representing approximately 80% of all acute ischemic cerebrovascular events (8), and in their prevention (9), which is very important since repeated strokes occur in 10–20% of patients within 3 months after the first stroke (1).

Antiplatelet agents are usually preferred over anticoagulant agents due to their connection with lower rates of intracranial hemorrhage and moderately lower global death rates. Antiplatelet monotherapy is usually favored over dual antiplatelet therapy (DAPT) because DAPT is often associated with severe bleeding complications (10). The guidelines of the American Heart Association and American Stroke Association suggest antiplatelet monotherapy with aspirin within 24–48 h of an acute ischemic stroke. The recommended doses range between 160 and 300 mg per day (11). Despite aspirin being suggested as therapy for acute ischemic stroke, it does not succeed in preventing platelet aggregation in 5–55% of patients (12). Nevertheless, the administration of DAPT immediately following a minor ischemic stroke (NIHSS score of ≤ 3) or high-risk TIA (ABCD2 score of ≥4) may be beneficial and outweigh the risks in patients with acute minor ischemic stroke or high-risk TIA. In this respect, the guidelines suggest 21-day treatment with aspirin and clopidogrel to be started within 24 h of symptoms onset with minor stroke. However, this combination should not be administered immediately after intravenous thrombolysis (IVT) (11).

Furthermore, two independent multicenter, randomized, double-blind, placebo-controlled trials have established the efficacy of short-term DAPT to prevent recurrent ischemic stroke in patients with minor stroke or high-risk TIA. The CHANCE trial (Clopidogrel in High-Risk Patients with Acute Non-disabling Cerebrovascular Events) in the Chinese population demonstrated a 32% reduction in recurrent stroke at 90 days (ischemic or hemorrhagic) with no increase in major bleeding (13). In the POINT trial (Platelet-Oriented Inhibition in New TIA and Minor Ischemic Stroke), conducted in North America, Europe, Australia, and New Zealand, up to 90 days after the index event, DAPT was associated with a 28% reduction in ischemic stroke (which was higher during the first 30 days of treatment) but was also associated with a higher number of major hemorrhages (14). However, in both trials (CHANCE and POINT), the loading dose of clopidogrel was given before the initiation of DAPT. In addition, researchers have also found that DAPT (aspirin with clopidogrel) may be ineffective in 5–30% of patients with percutaneous coronary interventions, mostly due to clopidogrel resistance. This number increases to 66% in patients with neuro-interventional events (15). Recently, Steffel et al. (16) discovered that rivaroxaban plus aspirin was associated with fewer adverse cardiovascular events but more major bleeding events than aspirin alone.

To date, clinical studies have provided evidence about four antiplatelet agents: aspirin, aspirin-dipyridamole, clopidogrel, and ticagrelor (9), which have also been widely described in clinical practice guidelines (11).

The purpose of this mini-review is therefore to provide an update on the main antiplatelet agents that have been successfully used in the secondary prevention of non-cardioembolic ischemic stroke and TIA, as well as to explore whether novel and effective antiplatelet agents and strategies have occurred in the secondary prevention of non-cardioembolic ischemic stroke and TIA.

## Methods and Materials

The authors of this mini-review conducted a literature search of peer-reviewed English language research articles listed in PubMed. The articles were searched using the following keywords: *antiplatelet therapy* AND *stroke, antiplatelet therapy* AND *ischemic stroke, antiplatelet agents* AND *ischemic stroke, antiplatelet therapy* AND *transient ischemic attack, antiplatelet agents* AND *transient ischemic attack*. The search was performed for studies published between January 1, 2015, and August 31, 2020, because several review studies on this topic summarized the literature prior to this period [cf. (17–19)]. The authors also conducted a backward search, i.e., they searched the references of the studies identified for relevant research studies that could have been missed during the PubMed searches. Only randomized controlled studies were included in the final analysis and evaluation.

## Results

Altogether, 11 randomized controlled trials (RCTs) were detected in PubMed and during the backward searches of the reference lists of the selected studies. Four studies originated in Japan, two in China, one in the UK, one in the USA, and one in Thailand, and two were multinational RCTs. The number of patients ranged from 21 to 13,199. The intervention period lasted from 1 week to 4 years. In all studies, aspirin was used either alone or with another antiplatelet drug. The most common were clopidogrel and cilostazol. The remaining antiplatelet drugs included in the studies were ticagrelor and dipyridamole. Cilostazol was exclusively used in studies of Asian origin, while clopidogrel was used across different continents. Table 1 below provides the basic characteristics of these drugs apart from the use of dipyridamole, which is now considered to be obsolete.

**Table 1:** Antithrombotic agents: platelet aggregation inhibitors (B01AC) (20, 21).

**Group of drugs**	**Antithrombotic agents: platelet aggregation inhibitors excl. heparin**			
Active agent	acetylsalicylic acid	clopidogrel	cilostazol	ticagrelor
ATC classification	B01AC06	B01AC04	B01AC23	B01AC24
Mechanism of action	Irreversible inhibition of COX-1 cyclooxygenase, thereby preventing the synthesis of thromboxane A2 in platelets and inhibiting their aggregation.	Irreversible selective inhibition of ADP binding to the platelet P2Y12 receptor and subsequent ADP-mediated activation of the GPIIb/IIIa glycoprotein complex, thereby inhibiting platelet aggregation.	Reversible inhibition of platelet aggregation.	Direct selective reversible antagonism of the P2Y12 receptor, which prevents platelet activation and aggregation
Active compound	Salicylic acid	SR26334	–	–
Half-time (h)	2–3 h (low doses)	8 h	10.5 h	1.5 h
Tmax (h)	From 10–20 min (acetylsalicylic acid) to 120 min (total salicylate)	30–60 min	–	2–4 h
Dose per day according to WHO (1)	1 tablet independent of strength (2)	75 mg p.o.	200 mg p.o.	180 mg p.o.
Most common side effects	Stomach pain, nausea, vomiting, diarrhea, GIT microhemorrhage, skin reactions.	Gastrointestinal bleeding, diarrhea, abdominal pain, dyspepsia, hematomas, epistaxis.	Headache, diarrhea, abnormal stools.	Bleeding and shortness of breath.

*(1) The DDDs are based on prophylaxis for thrombosis. (2) The DDD of acetylsalicylic acid is given as 1 tablet independent of tablet strength. This is due to the great variations between different countries in the dosages/strengths recommended for the prophylaxis of thrombosis*.

Furthermore, the use of individual antiplatelet agents and their combinations in the detected studies are described below.

*Monotherapy*: A recent study states that dual therapy is more beneficial than monotherapy. This corresponds to the fact that in the literature in recent years, we found very few studies of monotherapy. Amarenco et al. (7) demonstrated the superiority of ticagrelor (6.7%; *p* = 0.017) over aspirin (9.6%) in the prevention of stroke, myocardial infarction, and death after aspiration (9.6%) in patients with acute ischemic stroke or a transient ischemic attack at 90 days (HR 0.68; 95% CI 0.53–0.88; *p* = 0.003). Haungsaithong et al. (22) evaluated changes in mean platelet volume (MPV) after the use of four antiplatelet drugs (aspirin, clopidogrel, aspirin plus dipyridamole and cilostazol) in patients with acute non-cardioembolic ischemic stroke to assess the effect of antiplatelet therapy and MPV on the stroke outcome. The authors concluded that after 4 weeks of therapy, MPV was reduced, as well as the NIHSS score, but only clopidogrel reduced this score with statistical significance (*p* = 0.003).

Dual therapy: aspirin plus clopidogrel—this combination is the most common in the literature. Jing et al. (23) divided patients into three research subgroups (those with multiple acute infarctions, single acute infarctions and no acute infarctions) and compared the effect of dual therapy and monotherapy in these groups in terms of recurrent stroke. In the group with multiple acute infarctions, recurrent stroke occurred in the following percentage of patients: 10.1 (dual therapy) and 18.1% (aspirin alone), i.e., HR, 0.5; 95% CI, 0.3–0.96 (*p* = 0.04). In the group with single acute infarctions, no difference between dual therapy and aspirin alone was observed, i.e., 8.9 vs. 8.5% (HR, 1.1; 95% CI, 0.6–2.0; *p* = 0.71). For the no acute infarctions group, the results were as follows: 2.6 (dual therapy) vs. 1.4% (aspirin alone), i.e., HR, 1.7; 95% CI, 0.3–11.1 (*p* = 0.56). The authors concluded that dual clopidogrel and aspirin therapy appears to have the most significant clinical benefit for patients with multiple acute infarctions. Johnston et al. (14), based on a clinical study, reported that dual therapy vs. aspirin alone was associated with a lower risk of a severe ischemic event of 5.0 vs. 6.5%, i.e., HR 0.75; 95% CI, 0.59–0.95 (*p* = 0.02). He et al. (24) examined a group of patients with minor stroke or transient ischemic attack (TIA) to compare dual therapy vs. aspirin monotherapy for neurological deterioration, recurrent stroke, or stroke development in patients with a TIA within 14 days of admission. During the 2-week period, worsening of stroke occurred in nine patients in the dual therapy group and 19 patients in the monotherapy group. Stroke occurred after the TIA in one patient in the dual therapy group and in three patients in the monotherapy group. The authors concluded that early dual therapy can reduce neurological deterioration in patients with acute ischemic stroke as compared to monotherapy.

Dual therapy: aspirin plus cilostazol—two current studies can be found in the literature for this combination. Ohnuki et al. (12) found no significant differences in platelet aggregation, platelet activation, or endothelial biomarker levels in patients receiving dual therapy compared to the aspirin group. Aoki et al. (25) reported no significant differences between dual therapy and aspirin alone. The aim of this study was to determine the effectiveness of both therapies in patients with non-cardioembolic stroke within 48 h of the onset of symptoms. The treatment was evaluated as safe but failed to reduce the rate of short-term neurological worsening.

Dual therapy: aspirin plus ticagrelor—Amarenco et al. (26) evaluated the combination of ticagrelor and aspirin (as opposed to aspirin alone) in stroke prevention. The main endpoint was the time to stroke (progression of the event or a new stroke) or death within 30 days. Disabling stroke was defined by a modified Rankin Scale score (mRS) >1. A score of mRS > 1 occurred in the following percentage of patients: 4.0 (dual therapy) vs. 4.7% (aspirin alone), i.e., HR, 0.83; 95% CI, 0.69–0.99 (*p* = 0.04). Based on this study, it appears that ticagrelor added to aspirin was superior to aspirin alone in preventing disabling stroke or death at 30 days and reduced the total burden of disability owing to ischemic stroke recurrence.

The key findings reveal that DAPT appears to be safe and efficient in decreasing the risk of multiple ischemic strokes, particularly in the long term and with larger sample sizes (27), as well as being more effective than monotherapy in the early stages of acute ischemic stroke (24). This has also been confirmed by the most recent study by Amarenco et al. (26), in which patients with TIA and minor ischemic stroke received ticagrelor in combination with aspirin to prevent disabling stroke or death at 30 days. The results showed that DAPT was more efficient than aspirin alone in decreasing the total burden of disability due to ischemic stroke recurrence. However, the results differed in the risk of hemorrhage. While Jing et al. (23) and Toyoda et al. (27) confirmed a reduced risk of severe bleeding after 3 months, Johnston et al. (14) found the opposite result. Furthermore, the results indicated that the triple combination had no gains with respect to the occurrence and severity of recurrent stroke but actually had a higher rate of hemorrhage (28).

## Discussion

Our review of the literature revealed that aspirin is a reliable antiplatelet agent in the secondary prevention of acute non-cardioembolic ischemic stroke and TIA and that it is the only drug that has received a class 1A recommendation (11). However, Amarenco et al. (7), within the SOCRATES project, indicated that if ticagrelor was administered within 24 h of symptom onset, it would be more effective than aspirin in preventing stroke, myocardial infarction, or death at 90 days in patients with acute ischemic stroke or a transient ischemic attack when associated with potentially symptomatic ipsilateral atherosclerotic stenosis. Their findings also showed that there were no differences in the rate of life-threatening bleeding or major or minor bleeding episodes in patients with ipsilateral stenosis in the ticagrelor group compared with the aspirin group.

Haungsaithong et al. (22) reported that clopidogrel considerably decreased the NIHSS score (*p* = 0.003), and it resulted in the greatest reduction in MPV compared with the others. However, their study was quite small and followed the patients for only 4 weeks. Nevertheless, their findings were also confirmed by Paciaroni et al. (29) in their recent meta-analysis, in which they revealed smaller risks of severe undesirable cardiovascular or cerebrovascular episodes, recurrent stroke, and bleeding episodes for clopidogrel monotherapy compared to aspirin. These results confirmed the clinical benefits of antiplatelet monotherapy with clopidogrel over aspirin for secondary prevention in patients with a recent ischemic stroke.

In secondary prevention, dual antiplatelet therapy appears to be the best solution. A combination of aspirin and clopidogrel seems to be beneficial not only shortly (24 h to 1 week) after an acute non-cardioembolic stroke, most often a minor ischemic stroke, or a high-risk TIA (24) but also throughout the first 90 days after the event (14, 23, 27). These findings have also been confirmed by other studies (30, 31). Rahman et al. (30) stated that DAPT with aspirin and clopidogrel significantly reduced the risk of recurrent IS in the short-term (RR, 0.53; 95% CI, 0.37–0.78) and intermediate-term (RR, 0.72; 95% CI, 0.58–0.90). In addition, their findings also proved that intermediate-term (RR, 2.58; 95% CI, 1.19–5.60) and long-term (RR, 1.87; 95% CI, 1.36–2.56) aspirin and clopidogrel regimens significantly increased the risk of major bleeding compared to short-term aspirin and clopidogrel (RR, 1.82; 95% CI, 0.91–3.62), as revealed by Johnston et al. (14). The same findings were reported by Greving et al. (32). In their meta-analysis, they stated that a combination of clopidogrel and aspirin is beneficial for long-term secondary prevention after a non-cardioembolic stroke or transient ischemic attack, regardless of the patient characteristics. However, this combination is connected with a considerably higher risk of major bleeding than other DAPTs. Hao et al. (33) further found that DAPT with aspirin and clopidogrel administered within 24 h after high-risk TIA or minor ischemic stroke decreases the incidence of subsequent stroke by ~20 in 1,000 people, with a possible increase in moderate to severe bleeding of 2 per 1,000 population.

Furthermore, the evaluation of the detected trial (28) revealed that triplet antiplatelet therapy did not have any superiority over DAPT regarding the recurrence of strokes. In contrast, the rate of hemorrhage was much higher in these patients than in those who received fewer medications.

We also found that monotherapies with aspirin or ticagrelor and DAPT with aspirin and clopidogrel are mainly used in western Europe and the USA, while cilostazol, either alone or combined with aspirin, is predominantly used in Asia [cf. (34)]. However, the experts assume that there is no reason to test cilostazol in Europe and the USA since it has a good safety profile and seems safer than aspirin and is probably safer than other antithrombotic drugs in terms of reducing bleeding complications, especially hemorrhagic strokes.

The limitations of the included studies include their different sample sizes, various lengths of intervention periods, sometimes slightly different dosages of antiplatelet drugs, and an absence of sufficiently long follow-up periods in some studies.

Overall, the findings reveal that aspirin is a reliable antiplatelet agent in the secondary prevention of acute non-cardioembolic ischemic stroke and TIA. Nevertheless, currently, there are also other agents, i.e., ticagrelor, clopidogrel, and cilostazol, that can be applied. In addition, the results indicate that time is significant not only in severe stroke but also in non-severe stroke and TIA, which suggests that antiplatelet therapy should be administered within 24 h after the first symptoms (after 24 h in patients treated with IVT) because early treatment can lead to an improvement in neurological outcomes and a decrease in the risk of early subsequent stroke.

Future research should focus on identifying more effective drugs that could be developed for use in monotherapy because dual therapy only increases the rate of adverse events related to polypharmacy. In addition, these novel molecules could increase the risk of excessive bleeding. In fact, currently, a few studies are being conducted on this topic (Table 1).

## Author Contributions

MV, BK, MN, and RH equally contributed to the whole concept of the article, its methodology, data processing, and drafting and revision. All authors agreed with this final version of the manuscript.

## Conflict of Interest

The authors declare that the research was conducted in the absence of any commercial or financial relationships that could be construed as a potential conflict of interest.
